# Supplementary material for: The perception of eye contact is associated with men’s need to belong, self-esteem, and loneliness
Source: PLoS One. 2025 Mar 31;20(3):e0319974. doi: 10.1371/journal.pone.0319974 (PMC11957351; doi:10.1371/journal.pone.0319974)
Supplement: S1 Table — (DOCX) [file pone.0319974.s001.docx]

S1 Table Means and standard deviations of attractiveness ratings as a function of face identity and participants’ gender, as well as the statistical results of pairwise comparisons between men and women for each identity’s attractiveness ratings*.

| Identity | Participants' gender | Mean | Std. Deviation | Sig. | 95% confidence interval for difference |
| --- | --- | --- | --- | --- | --- |
| F1 | Woman | 6.50 | 1.43 | 0.74 | [-0.61, 0.85] |
|  | Man | 6.38 | 1.35 |  |  |
| F2 | Woman | 6.23 | 1.41 | 0.88 | [-0.71, 0.84] |
|  | Man | 6.17 | 1.56 |  |  |
| M1 | Woman | 5.00 | 1.55 | 0.42 | [-0.46, 1.08] |
|  | Man | 4.69 | 1.39 |  |  |
| M2 | Woman | 5.20 | 1.92 | 0.99 | [-0.91, 0.90] |
|  | Man | 5.21 | 1.54 |  |  |

*In the face stimuli assessment task, participants were instructed to rate the attractiveness of each identity on a scale from 1 to 9. 1 represented that the person looked “not at all attractive”; 9 represented that the person looked “highly attractive”.
