# Supplementary material for: The perception of eye contact is associated with men’s need to belong, self-esteem, and loneliness
Source: PLoS One. 2025 Mar 31;20(3):e0319974. doi: 10.1371/journal.pone.0319974 (PMC11957351; doi:10.1371/journal.pone.0319974)
Supplement: Attachment — Submitted filename: Response to Reviewers.docx [file pone.0319974.s002.docx]

Manuscript ID: PONE-D-24-25863

Dear editor,

We thank you and the reviewers for all the constructive and helpful comments. They greatly helped us to improve the manuscript. Below, we address each of the reviewers’ concerns one by one. However, before doing so, we would like to clarify an issue and highlight a few minor changes. In Figure 2, the face images included were generated by compositing and do not depict a real person. Therefore, the PLOS ONE policy on papers that include identifying, or potentially identifying, personal information does not apply to our manuscript. To clarify it, we pointed it out in the section of *Stimuli and programming of the experiment* by adding a sentence, *“The face stimuli were four neutral faces (two females and two males) which were created using 3D animation software (Digital Art Zone 3D studio, Daz 3D) (*[*https://www.daz3d.com/*](https://www.daz3d.com/)*), thus the face images did not depict real people”* (Page 10, Lines 203-205). Additionally, we reviewed the reference list, ensuring the page numbers of cited articles and chapters are complete, and removed the URLs for DOIs.

Reviewer #1:

*I think it is a very good attempt. The authors have presented all the details in a very intelligent and engaging fashion. What I would suggest though is that if the title can be amended slightly so that it is improved. I would not like to see something in the title like "...in men but not in women".*

Thank you for the suggestion. As the study did not aim to make gender-based comparisons regarding the association between eye contact perception and individuals’ socioemotional characteristics, we revised the title to: “The perception of eye contact is associated with men’s need to belong, self-esteem, and loneliness”. This revised title highlights the key findings of our study.

Reviewer #2:

1) *Reviewer wants to know what the primary hypothesis of this study on the perception of eye contact and its associations is.*

In the revised version, we clarified the primary hypothesis of this study at the very beginning in section 1.3. “*Overview of the present study”* on Pages 8-9, Lines 170-178.

*“The primary research question was whether CoDG predicts NTB, self-esteem, and loneliness. We hypothesized that individuals with a narrow CoDG may have a high NTB, low self-esteem, and high loneliness. In addition, based on various perspectives in the research literature, we also investigated whether CoDG predicts self-esteem and loneliness via NTB (serial mediation models, Figure 1a-1b; moderated mediation models, Figure 1c-1d). We first investigated the relationship between CoDG and NTB. Because previous research has shown that there are gender differences in the CoDG width [31,32] as well as in NTB strength [33,50,51], we also explored whether there were gender differences in NTB and CoDG in the present sample of participants. If that would be the case, we would analyse the results separately for men and women”.*

2) *Reviewer wants to understand how the "cone of direct gaze" (CoDG) is defined in this study and why it is significant.*

We agree with the reviewer that it is important to explain how the “cone of direct gaze (CoDG)” was defined in the present study. Gamer and Hecht (2007) hypothesized that “there is a range of considerable width wherein a person feels looked at”, using the metaphor of a cone of direct gaze to describe this range of gaze directions perceived as eye contact by an observer. The definition of CoDG in this study aligns with this widely accepted descriptive concept.

However, CoDG can be measured using different methods, which may affect the estimation of its width (Linke & Horstmann, 2024). Thus, it is necessary to clarify the method used in the present study. Building on the work by Gibson and Pick (1963), participants were shown face images displaying varying gaze directions (ranging from 0˚ to 8˚ in an increment of 2˚) and asked to judge whether they were being looked at by responding with “yes” or “no” (Details of two alternative methods are available in Linke and Horstmann, 2024). Based on these dichotomous eye contact judgments and the continuous variation in gaze directions, a point of subjective equality (PSE)—the gaze direction with a 50% probability of being judged as a direct gaze or averted gaze—was calculated using a binary logistic regression model. The width of CoDG was then determined by multiplying PSE with two. Without this methodological clarification, readers might find the definition of CoDG unclear in Introduction. However, since the detailed calculation of CoDG width fits more appropriately in the method section, and the cone metaphor effectively conveys the concept of eye contact perception, we kept the definition brief in Introduction. In the revised version, we clarified this point and included a reminder directing readers to where CoDG calculation is explained in detail, in the manuscript on Page 3, Lines 54-56.

*“This has been investigated by measuring the cone of direct gaze (CoDG), a range of gaze directions slightly deviating from the direct gaze which observers accept as eye contact [3] (see section Data analysis for how CoDG width was calculated)”*.

3) *Reviewer wants to explore the main differences observed between men and women regarding CoDG and its psychological associations.*

The main difference is that, for men, CoDG width was correlated with NTB, self-esteem, and loneliness, whereas, for women, CoDG was not correlated with NTB, self-esteem, or loneliness. The specific values and significance are provided on Page 17, Lines 331-336 and in Table 2.

*4) Reviewer wants to clarify what specific tasks participants completed during the eye contact judgment experiment.*

As addressed in point 2), we presented face images displaying eleven gaze directions (left: 2˚, 4˚, 6˚, 8˚, 20˚; direct: 0˚; right: 2˚, 4˚, 6˚, 8˚, 20˚), which participants judged for making eye contact with them or not. Further details can be found on Page 11, Lines 226-238.

*5) Reviewer wants to understand how participants' levels of self-esteem, loneliness, and need to belong were measured.*

We measured the need to belong, self-esteem, and loneliness using the Need to Belong Scale (Leary et al., 2013), the Rosenberg Self-Esteem Scale (Rosenberg, 1965), and the UCLA Loneliness Scale (Russell, 1996), respectively. Participants completed these scales in the fixed order mentioned above. Instead of viewing all items at once, participants in our study were presented with one item each time, responding by pressing a number key. The items on each scale were presented in random order for each participant. Further details can be found on Pages 11-12, Lines 242-260.

*6) Reviewer wants to examine why certain participants were excluded from the final analysis and how data quality was ensured.*

To ensure data quality, we incorporated several attention check items into the experimental design and implemented strict data cleaning procedures.

Firstly, attention check items were included in the eye contact judgment task (16 attention-check trials among 160 test trials), the Need to Belong Scale (1 in 10), the Rosenberg Self-Esteem Scale (1 in 10), and the UCLA Loneliness Scale (2 in 20). Inspecting the responses to attention check items could reveal whether participants paid enough attention during the online tasks. In the eye contact judgment task, participants were excluded if their percentage of "yes" responses to catch trials (faces with gaze direction of 20˚) exceeded 50%. Similarly, in the questionnaire (composed of 40 items), participants were excluded if they failed more than two (out of four) attention check trials. In the revised version, this point was clarified on Pages 12-13, Lines 264-272:

“*In the eye contact judgment task, the participant was excluded if the percentage of “yes” responses to catch trials (faces with gaze direction of 20˚) was more than 50%. One participant who gave 87.5% “yes” responses to these catch trials was excluded. In the questionnaire (composed of 40 items), participants were excluded if they failed more than two (out of four) attention check trials. No participant was excluded due to attention checks in the questionnaire* (19 participants failed one attention check question, and 2 failed two). *Thus, 399 participants’ data were analyzed subsequently. In the subsequent analysis of gaze cone for the 399 participants, those whose data did not meet the criteria for calculating CoDG width were excluded, even if they had passed the attention check questions among the three scales*”.

For the final sample, we reported the average percentage of "yes" responses to catch trials in the eye contact judgment task on Page 14, Lines 294-297.

*“We also checked these participants’ responses on attention check trials (16 trials of 20˚ gaze direction), and the average error rate on the catch trials was 0.46%. Two participants made 2 erroneous responses, twenty-three participants made 1, and the remaining 342 participants made no erroneous responses”.*

Second, after removing attention check items, we applied a series of exclusion criteria when calculating CoDG width:

1 Pre-calculation exclusion: Participants were excluded if the percentage of “yes” responses to 8˚ gaze directions (leftward and rightward averted gaze) exceeded 50%, or if the percentage of “yes” responses to direct gaze (0˚) was below 50%. Additionally, participants who self-reported technical issues that could have affected their results were excluded.

2 post-calculation exclusion: The goodness of fit for binary logistic regression models used to calculate CoDG width was assessed by examining deviance residuals. Participants with deviance residuals exceeding their corresponding degrees of freedom, indicating a poor model fit, were excluded.

3 Manual inspection: CoDG width values were manually inspected for abnormalities, such as negative values.

Details about participant exclusions based on these criteria can be found on Pages 13-14, Lines 273-278 and Lines 288-298.

*7) Reviewer wants to investigate the relationship between CoDG width and loneliness among men.*

Among men, the results showed a negative correlation between CoDG width and loneliness level (Page 17, Line 333 and Table 2), suggesting that men with narrower CoDG reported higher loneliness. In social interactions, an averted gaze may signal avoidance-oriented motivation by the sender (Adam & Kleck, 2003, 2005) and activate a receiver’s motivation tendency of avoidance (Hietanen et al., 2008; Uusberg et al., 2015). Thus, a narrow CoDG, reflecting a tendency to interpret slight gaze deviations as averted gaze, may have adverse effects on the formation and maintenance of social bonds. This difficulty in initiating or sustaining desired social relationships can lead to social isolation (loneliness). A more detailed discussion of this point can be found on Pages 22-23, Lines 425-436.

*8) Reviewer wants to analyze how NTB mediates the relationship between CoDG width and self-esteem.*

In the serial mediation model, CoDG width predicted loneliness through the serial mediation of NTB and self-esteem, with NTB also mediating the association between CoDG width and self-esteem in men (Page 19, Lines 352-353). We suggested that a narrower CoDG may decrease opportunities for forming and maintaining relationships among men. The lack of regular contact or meaningful bonds with others cannot fully satisfy NTB. Further, a high NTB may increase concerns regarding relationship devaluation or lead to the eventuality of unsatisfying relationships, which is reflected in low self-esteem. A more detailed discussion is provided on Pages 24-25, Lines 467-482.

*9) Reviewer wants to know why CoDG was not associated with NTB, self-esteem, and loneliness among women.*

On Pages 26-27, Lines 499-521, we discuss why CoDG was not associated with NTB, self-esteem, and loneliness among women. We hypothesized that the joint effects of CoDG width and NTB would predict individuals’ self-esteem and loneliness levels. However, the absence of an association between CoDG and NTB may explain why CoDG width cannot serve as a predictor for self-esteem or loneliness. To address the reviewer’s question (also our own), we explored possible reasons for the gender difference in the CoDG-NTB association. Specifically, a negative correlation was observed between CoDG and NTB among men, whereas no such association was found for women. While people are fundamentally motivated to establish and sustain relationships regardless of gender, differences exist in how belongingness is pursued (Baumeister & Sommer, 1997) and how social relationships are mentally represented (Eck et al., 2017; Foels & Tomcho, 2009). Men may be oriented to large groups of shallow relationships and see social groups as overall categories (e.g., family or college). Therefore, CoDG width likely affects whether they can build broad social networks. In men, narrower CoDG together with the representation of social relationships as categories may make it more difficult in forming dyadic and meaningful social interactions. In contrast, women’s sociality is primarily oriented toward intimate and close relationships, and they see social relationships as dyadic bonds (e.g., sister or classmate). As such, judging nonsignificant others as not making eye contact does not affect their sense of belonging. Women just need to recognize potential dyadic relationships first and engage in meaningful interactions with specific individuals. Thus, their NTB can be met by social interactions of high quality, regardless of their CoDG width.

*10) Reviewer wants to understand how the findings explain the gender-specific effects of CoDG width on NTB and loneliness.*

If we understand this comment from the reviewer correctly, we want to raise two aspects in our response. On one hand, in this study, we did not find gender differences in loneliness. Although we observed gender differences in CoDG width and NTB strength, we cannot conclude that the gender difference in NTB is the effect of the gender difference in CoDG width. On the other hand, a negative correlation was observed between CoDG and NTB among men, whereas no such association was found for women. In men, narrower CoDG predicts higher loneliness through the link of higher NTB and lower self-esteem. However, in women, this study did not show the predictive effect of CoDG width on self-esteem or loneliness perhaps due to the absence of association between CoDG and NTB. Then, we can return to comment 9) to think about the gender difference in the CoDG-NTB association. As with how men’s CoDG width together with NTB exert influences on loneliness, the detailed discussion can be found on Pages 24-25, Lines 467-482.

*11) Reviewer wants to assess what theoretical explanations are proposed for why men and women experience different social dynamics based on CoDG.*

As addressed in our response to comment 9), with a more detailed discussion on Pages 26-27, Lines 499-521, we proposed that gender differences exist how belongingness is pursued (Baumeister & Sommer, 1997) and how social relationships are mentally represented (Eck et al., 2017; Foels & Tomcho, 2009). These theories may help explain why men and women experience different social dynamics based on CoDG. However, we want to emphasize that these interpretations are not exclusive and that there may be other theories accounting for our findings. Since this study aimed to tentatively explore whether there would be gender difference and what the gender difference would be in the use of CoDG width to predict socioemotional characteristics, we did not test any theories regarding the mechanisms underlying the social dynamics associated with CoDG.

*12) Reviewer wants to identify the broader implications of this research for understanding social cognition and interpersonal relationships.*

The broader theoretical and practical implications of this research can be found on Page 28, Lines 547-559.

*13) Reviewer wants to consider the limitations of using the NTBS questionnaire in studying the need to belong.*

The construct of the need to belong may be multifaceted, encompassing but not limited to a growth orientation (an approach-focused motive directing toward interpersonal actualization) and a deficit-reduction orientation (an avoidance-focused motive directing toward interpersonal deficit reduction or repair) (Lavigne et al., 2014). Notably, the NTBS primarily measures the avoidance-oriented aspect (Pillow et al., 2015). On Page 27, Lines 529-535, we acknowledged that our study might be limited by assessing the need to belong only via the NTBS questionnaire.

*14) Reviewer wants to analyze how the study addresses potential cultural influences on the observed patterns.*

On Pages 27-28, Lines 536-545, we tentatively analyzed the potential cultural influences on the observed patterns. However, since the study did not include a sample from another cultural background, we chose to avoid extensive speculation about cultural influences.

*15) Reviewer wants to know what avenues for future research are suggested by the findings, particularly regarding gender differences and CoDG.*

We appreciate the reviewer’s encouragement for further exploration. This study aimed to investigate how individuals’ eye contact perception predicts their socioemotional characteristics. In gaze perception literature, eye contact judgment, as measured by CoDG width, has been shown to be influenced by contextual factors related to both observers (e.g., social anxiety; Gamer et al., 2011; Harbort et al., 2013; Jun et al., 2013) and gazers (e.g., facial emotions and attractiveness; Ewbank et al., 2009; Kloth et al., 2011; Lobmaier & Perrett, 2011; Lobmaier et al., 2008). One possible avenue for future research could involve incorporating situational factors to deepen the understanding of the relationship between CoDG and socioemotional characteristics. Additionally, as noted on Page 29, Lines 566-568, there is a need to develop a theoretical framework that addresses how, in women, eye contact perception, in association with socioemotional characteristics influences social interactions. Another promising direction would be to use a network analysis approach to map the relationships among women’s facial information processing, personality traits, and social relationships. In the revised version, we added the following sentences on Page 29, Lines 568-575:

“*One potential avenue is to identify key factors shaping the association between CoDG and socioemotional characteristics, such as factors related to observers (e.g., personality traits) and gazers (e.g., facial features or other information relevant to social desirability). Additionally, a network analysis approach, well-suited for examining the structures of complex relations, could help researchers explore the dynamics in a network involving women’s eye contact perception, personality traits (e.g., Big Five traits), and the quality of their social interactions (e.g., general satisfaction in social relationships, satisfaction of romantic relationships, and friendships)*”.

References

Adams Jr, R. B., & Kleck, R. E. (2003). Perceived gaze direction and the processing of facial displays of emotion. *Psychological science*, *14*(6), 644-647. <https://doi.org/10.1046/j.0956-7976.2003.psci_1479.x>

Adams Jr, R. B., & Kleck, R. E. (2005). Effects of direct and averted gaze on the perception of facially communicated emotion. *Emotion*, *5*(1), 3. [https://doi.org/10.1037/1528-3542.5.1.3](https://psycnet.apa.org/doi/10.1037/1528-3542.5.1.3)

Baumeister, R. F., & Sommer, K. L. (1997). What do men want? Gender differences and two spheres of belongingness: Comment on Cross and Madson (1997). *Psychological Bulletin*, *122*(1), 38-44. <https://doi.org/10.1037/1089-2680.8.2.111>

Eck, J., Schoel, C., & Greifeneder, R. (2017). Belonging to a majority reduces the immediate need threat from ostracism in individuals with a high need to belong. *European Journal of Social Psychology*, *47*(3), 273–288. https://doi.org/10.1002/ejsp.2233

Ewbank, M. P., Jennings, C., & Calder, A. J. (2009). Why are you angry with me? Facial expressions of threat influence perception of gaze direction. *Journal of Vision*, *9*(12), 16–16. <https://doi.org/10.1167/9.12.16>

Foels, R., & Tomcho, T. J. (2009). Gender differences in interdependent self-construals: It's not the type of group, it's the way you see it. *Self and Identity*, *8*(4), 396-417. <https://doi.org/10.1080/15298860802391470>

Gamer, M., & Hecht, H. (2007). Are you looking at me? Measuring the cone of gaze. *Journal of Experimental Psychology: Human Perception and Performance*, *33*(3), 705–715. <https://doi.org/10.1037/0096-1523.33.3.705>

Gamer, M., Hecht, H., Seipp, N., & Hiller, W. (2011). Who is looking at me? The cone of gaze widens in social phobia. *Cognition and Emotion*, *25*(4), 756-764. <https://doi.org/10.1080/02699931.2010.503117>

Gibson, J. J., & Pick, A. D. (1963). Perception of another person’s looking behavior. The American Journal of Psychology, 76(3), 386–394. <https://doi.org/10.2307/1419779>

Harbort, J., Witthöft, M., Spiegel, J., Nick, K., & Hecht, H. (2013). The widening of the gaze cone in patients with social anxiety disorder and its normalization after CBT. *Behaviour Research and Therapy*, *51*(7), 359–367. https://doi.org/10.1016/j.brat.2013.03.009

Hietanen, J. K., Leppänen, J. M., Peltola, M. J., Linna-aho, K., & Ruuhiala, H. J. (2008). Seeing direct and averted gaze activates the approach–avoidance motivational brain systems. *Neuropsychologia*, *46*(9), 2423–2430. <https://doi.org/10.1016/j.neuropsychologia.2008.02.029>

Jun, Y. Y., Mareschal, I., Clifford, C. W. G., & Dadds, M. R. (2013). Cone of direct gaze as a marker of social anxiety in males. *Psychiatry Research*, *210*(1), 193–198. https://doi.org/10.1016/j.psychres.2013.05.020

Kloth, N., Altmann, C. S., & Schweinberger, S. R. (2011). Facial attractiveness biases the perception of eye contact. *Quarterly journal of experimental psychology*, *64*(10), 1906-1918. <https://doi.org/10.1080/17470218.2011.587254>

Lavigne, G. L., Vallerand, R. J., & Crevier-Braud, L. (2011). The fundamental need to belong: On the distinction between growth and deficit-reduction orientations. *Personality and social psychology bulletin*, *37*(9), 1185-1201. <https://doi.org/10.1177/0146167211405995>

Leary, M. R., Kelly, K. M., Cottrell, C. A., & Schreindorfer, L. S. (2013). Construct validity of the Need to Belong Scale: Mapping the nomological network. *Journal of Personality Assessment*, *95*(6), 610–624. https://doi.org/10.1080/00223891.2013.819511

Linke, L., & Horstmann, G. (2024). New task–new results? How the gaze cone is influenced by the method of measurement. *Attention, Perception, & Psychophysics*, 1-16. <https://doi.org/10.3758/s13414-024-02884-9>

Lobmaier, J. S., & Perrett, D. I. (2011). The world smiles at me: Self-referential positivity bias when interpreting direction of attention. *Cognition & Emotion*, *25*(2), 334–341. https://doi.org/10.1080/02699931003794557

Lobmaier, J. S., Tiddeman, B. P., & Perrett, D. I. (2008). Emotional expression modulates perceived gaze direction. *Emotion*, *8*(4), 573–577. <https://doi.org/10.1037/1528-3542.8.4.573>

Pillow, D. R., Malone, G. P., & Hale, W. J. (2015). The need to belong and its association with fully satisfying relationships: A tale of two measures. *Personality and individual differences*, *74*, 259-264. <https://doi.org/10.1016/j.paid.2014.10.031>

Rosenberg, M. (1965). Society and the adolescent self-image. Princeton, NJ: Princeton University Press.

Russell, D. (1996). UCLA Loneliness Scale (Version 3): Reliability, validity, and factor structure. *Journal of Personality Assessment, 66*, 20-40. http://dx.doi.org/10.1207/s15327752jpa6601_2

Uusberg, H., Allik, J., & Hietanen, J. K. (2015). Eye contact reveals a relationship between Neuroticism and anterior EEG asymmetry. Neuropsychologia, 73, 161–168. https://[doi:10.1016/j.neuropsychologia.2015.05.008](http://dx.doi.org/10.1016/j.neuropsychologia.2015.05.008)
